# The impact of a preanesthesia assessment clinic on scheduled elective case cancelations at a Thai university hospital

**DOI:** 10.1097/MD.0000000000034823

**Published:** 2023-08-25

**Authors:** Wariya Vongchaiudomchoke, Pitchapa Wongcharoen, Mingkwan Wongyingsinn

**Affiliations:** a Department of Anesthesiology, Faculty of Medicine Siriraj Hospital, Mahidol University, Bangkok, Thailand.

**Keywords:** case cancelation, preoperative care, reason for cancelation

## Abstract

Elective surgical case cancelations negatively impact healthcare systems and patient dissatisfaction. Preanesthesia assessment clinics (PACs) have been established in many countries to facilitate preoperative medical optimization. However, their benefits for elective procedure cancelations in Thailand have not been formally assessed. This study evaluated the impact of a PAC on scheduled elective surgical case cancelations at a Thai university hospital. A retrospective cross-sectional study was conducted for the period covering from May 2016 to April 2017. We included all scheduled elective surgical cases at Siriraj Hospital, Thailand, canceled on the day of surgery. The cancelation incidences of patients attending and not attending the PAC were compared. Cancellation reasons were categorized as “patient issue,” “hospital-facility issue,” “surgeon issue,” “anesthesiologist issue,” “medical condition,” and “miscellaneous.” The PAC patients’ reasons were rigorously explored to determine their preventability. There were 30,351 scheduled elective procedures during the study period. The case-cancelation incidences were 0.9% (95% confidence interval [CI], 0.7–1.2%) for patients visiting the PAC and 5.9% (95% CI, 5.6–6.3%) for those who did not. Medical conditions were the most common reason for cancelation for non-PAC patients (27.3%), whereas hospital-facility issues were the most frequent for PAC patients (43.8%). The cancelation rate for patient issues was significantly lower in the PAC group (4.2% vs 20.7%; *P* < .05). Thirty-one (64.6%) of the PAC patients’ cancelations were potentially preventable. Of the 15 PAC patient cancelations related to medical conditions, 12 were for patients with a history of acute illness and were determined to be nonpreventable. Visiting the PAC was significantly associated with a decreased elective-case cancelation rate. Cancellations were most frequently related to hospital-facility issues for patients visiting the PAC and medical conditions for those who did not. Some PAC patient cancelations for medical conditions involved unpreventable acute patient illnesses. Clinical Trials.gov (NCT02816281).

## 1. Introduction

Elective surgical case cancelations significantly impact hospital resource utilization. The consequences of the cancelation or postponement of elective surgery include inefficient usage of operating rooms, economic losses, resource wastage, patient dissatisfaction, and treatment delays.^[1,2]^ The cancelation rate is a parameter for assessing the quality of patient care and hospital management systems.^[3]^ The literature reports cancelation rates ranging from 5% to 20%, varying with the definition of cancelation used and the institutional setting. The rates have also been found to correlate with countries’ national incomes.^[4–9]^ Three studies determined case cancelation rates as high as 66% to 74%, according to a meta-analysis published in 2020.^[4]^ In Asia, the rates have been reported to range between 5% and 18%.^[5,10–12]^ Although the reasons for cancelation varied considerably between studies, they were generally classified into 6 categories: “patient issue,” “surgeon issue,” “medical condition,” “hospital-facility issue,” “anesthesiologist issue,” and “miscellaneous” (Table 1).^[11,13]^

**Table 1:** Categorization of reasons for patient cancelations.

Category	Reason
Patient issue	Patient no-show; patient refusal; patient death
Hospital-facility issue	Case bump; improperly estimated case time; intensive care unit bed unavailability; blood product unavailability; equipment unavailability; hospital bed unavailability
Surgeon issue	Changed line of surgical management; inadequate preparation (e.g., inadequate bowel preparation); scheduling error; surgeon unavailability
Anesthesiologist issue	Inadequate nil per os status; anesthesiologist refusal; preoperative drug error
Medical condition	Cardiovascular problem (e.g., hypertension, shock, acute coronary syndrome); electrolyte imbalance; endocrine problem (e.g., hyperglycemic crisis, uncontrolled hyperthyroid); eye problem (e.g., hordeolum, conjunctivitis); fever–unspecified (body temperature more than 38°C); gastrointestinal tract problem (e.g., diarrhea); gynecological problem (e.g., menstruation); hematologic problem (e.g., anemia, thrombocytopenia, coagulopathy); neurologic problem (e.g., recent stroke, alteration of consciousness); respiratory tract problem (e.g., pneumonia, respiratory tract infection); skin infection (e.g., dermatophyte infection, lice infection); urogenital problems (e.g., urinary tract infection, acute kidney injury)
Miscellaneous	Health insurance problem; reason could not be determined from medical records; data unavailability

Preanesthesia assessment clinics (PACs) have generally been introduced to optimize medical conditions before surgery, improve patient care, and lower the perioperative care costs of patients undergoing surgery requiring an anesthesia service.^[14,15]^ PACs also improve hospital resource utilization by reducing preoperative consultations, unnecessary laboratory testing, and investigations. In addition, having patients visit a PAC is one of the approaches used to reduce scheduled elective procedure cancelations. This strategy has been shown to reduce the duration of hospital stays and enhance patient satisfaction.^[10,16]^

In Thailand, no studies have previously compared the outcomes of surgical patients who visited a PAC and those who did not. In particular, no investigation of the benefits of PACs has been carried out in a university hospital context, where consultation indications may be distinct from other hospital settings. Therefore, we initiated this retrospective study to assess the benefits of the PAC at a quaternary teaching hospital in Thailand. We hypothesized that patients who attended the PAC preoperatively would have a lower incidence of elective case cancelation on the day of surgery than those who did not. We hypothesized that this reduction would be particularly evident with cancelations related to medical conditions that could be prevented by appropriate preoperative evaluation and preparation by the PAC. The reasons for canceling cases with a history of PAC visits were also analyzed to assess the effectiveness of the PAC’s preoperative patient evaluations and preparations. This research followed the STROBE reporting guidelines for observational studies.

## 2. Materials and Methods

After Siriraj Institutional Review Board approval (SIRB410/2016), a retrospective data analysis of canceled elective surgical procedures at Siriraj Hospital was conducted. It covered May 1, 2016, to April 30, 2017. In this study, all scheduled elective surgical procedures during the study period were included. Exclusion criteria included emergency procedures, procedures not requiring an anesthesia service, specialties not consulting the PAC, and the redundant data. The number of missing data (if any) would be reported for each phase of the analysis.

### 2.1. Hospital setting

Siriraj Hospital is a university hospital and quaternary-care medical center. It has a capacity of approximately 2200 beds and 63 operating rooms for 16 surgical specialties. An anesthesiologist-initiated PAC, named Siriraj Preanesthesia Assessment Center, was established at the hospital in 2008. To maximize accessibility and convenience, the clinic is located in the same outpatient department as other surgical clinics. After deciding to perform surgery, surgeons may refer their patients for evaluation by the PAC.

The PAC service encompasses a patient interview; physical examination; review of previous medical, surgical, and anesthetic problems; verification of current medication use; advice on obtaining and reviewing preoperative tests; and risk reduction through appropriate interventions and consultations. Consultant anesthesiologists in the PAC are primarily responsible for policy administration, risk assessment, and perioperative management decisions. Anesthesiology and surgery residents must complete a clinical rotation through the PAC as part of their preoperative evaluation and preparation training.

According to our hospital’s policy, patients with an American Society of Anesthesiologists (ASA) physical status of III or higher should be assessed and optimized at the PAC within 3 months before the scheduled date of their surgery. However, attending physicians can decide whether to refer patients to the PAC for preoperative preparation. Patients with an ASA physical status of I or II are also indicated for PAC consultation if they:

- have an abnormal physical examination or laboratory testing;
- are undergoing major surgery (any invasive operative procedure lasting more than 3 hours or posing a life-threatening risk); or
- have other indications (such as a history of difficult intubation or an adverse anesthesia event).

**Figure 1.**
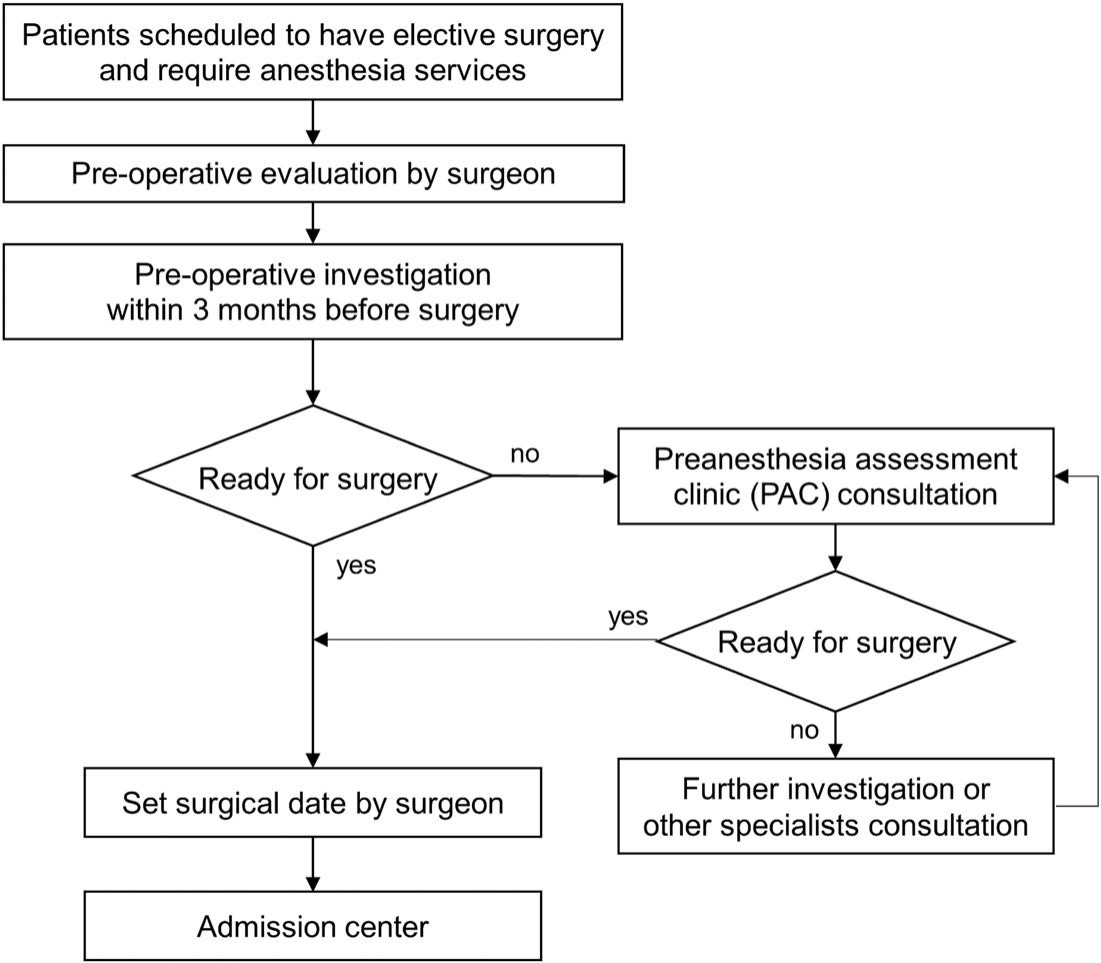
The flow of the preanesthesia clinic consultation process.

The flow of the PAC consultation process is illustrated in Figure 1.

Regarding our institution’s practices, gastrointestinal endoscopic patients are evaluated by consultant anesthesiologists at the Siriraj Gastrointestinal Scope Center and are not referred to the PAC. Moreover, the clinic typically does not evaluate obstetrics, diagnostic and interventional radiology, percutaneous cardiac intervention, or psychiatric patients. The non-PAC patients are assessed and optimized by attending physicians themselves prior to admission. All patients, both PAC and non-PAC patients, are provided an updated pre-anesthesia evaluation by anesthesiologists when patients come to the hospital for surgery and anesthesia.

### 2.2. Data collection

For this investigation, “elective surgeries” were defined as any operations added to the hospital’s operating room schedule at least 1 day before the surgery date. In addition, the starting times of the elective cases were required to be during a 7-hour window (9 am to 4 pm) on weekdays. Emergency operations, procedures that did not require anesthesia, and specialties in which the patients were not referred to the PAC were excluded. “Canceled cases” were defined as elective procedures in the schedule that were later canceled on the day of surgery.

Details of all scheduled elective surgeries, including basic patient information and planned procedures, were collected from the hospital’s surgery scheduling system. For the cases included in this study, we verified the PAC visit information by confirming that the proposed procedures discussed during the PAC consultations corresponded with the details of the canceled operations. Information on the PAC visits was obtained from the anesthesiology department’s database.

The data on total scheduled case cancelations were retrieved from the anesthesiology department’s database and the hospital’s risk management division. The reasons for surgery cancelations were grouped into 6 categories: “patient issue,” “surgeon issue,” “medical condition,” “hospital-facility issue,” “anesthesiologist issue,” and “miscellaneous.” Definitions of each term are given in Table 1. Individual case cancelations were assigned to only 1 category.

For patients who attended the PAC, an additional analysis of each cancelation reason was performed, with each being classified as “potentially preventable” or “nonpreventable.” Potentially preventable reasons referred to cancelations that could have been avoided if existing procedures and instructions had been followed. The reasons encompassed patient no-shows; improperly estimated case times; unavailability of blood products, equipment, surgeons, and intensive care unit (ICU) or hospital beds; changed line of surgical management; inadequate preparation; scheduling errors; inadequate nil per os status; anesthesiologist refusals; preoperative drug errors; and health insurance problems. Nonpreventable reasons referred to cancelations that could not be avoided (patient refusal, patient death, and case bumps). Cancellations related to medical conditions and anesthesiologist issues were also examined to ascertain whether being serviced by the PAC could have prevented the surgery from being canceled.

### 2.3. Statistical analysis

The demographic variables were summarized using descriptive statistics. Categorical variables are reported as frequencies and percentages, while continuous variables are presented as the means and standard deviations or medians and interquartile ranges, depending on the data distribution. Categorical variables were compared using the chi-squared or Fisher’s exact test. A *P* value of .05 or less was considered to indicate statistical significance. Statistical analyses were performed using PASW Statistics for Windows, version 18.0 (SPSS Inc, Chicago, IL).

## 3. Results

### 3.1. Incidence of case cancelations

**Figure 2.**
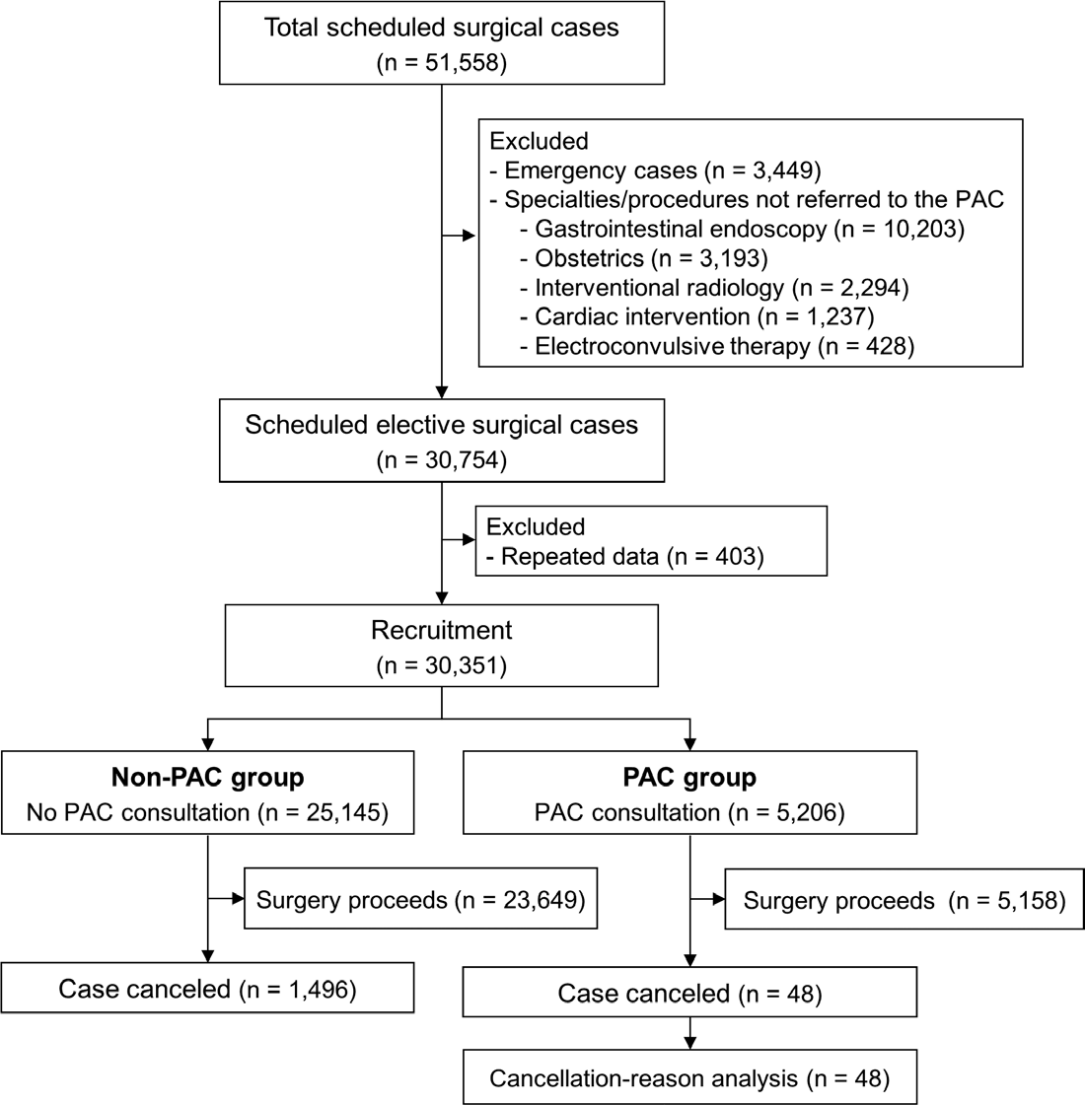
Flow of study patients. PAC = preanesthesia assessment clinic.

The flow of the study patients is illustrated in Figure 2. Our institution had 51,558 scheduled surgical procedures during the 1-year study period. We excluded emergency procedures, operations not requiring anesthesia, and specialties in which patients were not referred to the PAC. After removing redundant data, 30,351 scheduled elective cases remained and were enrolled in our study. Of those cases:

- A total of 5206 (17.1%) had a history of attending the PAC before surgery. These were termed “the PAC group.”
- A total of 25,145 (82.8%) did not have a history of attending the PAC (“the non-PAC group”).
- A total of 1544 were canceled on the proposed operative date. These case withdrawals gave an overall cancelation rate of 5.1% (95% CI, 4.8–5.3%).

Of the 1544 surgeries canceled on the scheduled operative date, the non-PAC group accounted for 1496 (cancelation rate, 5.9%; 95% CI, 5.6–6.3%), whereas there were 48 cases from the PAC group (cancelation rate, 0.9%; 95% CI, 0.7–1.2%). Patients attending the PAC had a 5-percentage-point lower cancelation rate (95% CI, 4.6–5.4%) than those who did not attend the clinic (*P* < .001).

The demographic data relating to the canceled cases are detailed in Table 2. Most patients were 18 to 65 years old, and nearly half of the patients in the non-PAC and PAC groups were men. The top 3 surgical specialties in the non-PAC group were orthopedics (17.0%), plastic and reconstruction surgery (15.6%), and otorhinolaryngology (14.6%). The top specialty in the PAC group was vascular surgery (18.8%), while orthopedics, ophthalmology, and urologic surgery jointly ranked second (14.6% each).

**Table 2:** Demographics of canceled cases.

Characteristics	Non-PAC group (n = 1496)		PAC group (n = 48)		*P* value
	Number	%	Number	%	
Sex, male	715	47.8	24	50	.771
Age					
<18	254	17.0	4	8.3	.023
18–65	857	57.3	25	52.1	
>65	344	23.0	19	39.6	
Hospital status, inpatient	1036	69.3	43	89.6	.002
Surgical specialty					
Orthopedics	255	17.0	7	14.6	<.001
Plastic and reconstructive surgery	234	15.6	1	2.1	
Otorhinolaryngology	218	14.6	3	6.3	
Ophthalmology	137	9.2	7	14.6	
Cardiothoracic surgery	107	7.2	3	6.3	
General surgery	98	6.6	4	8.3	
Neurological surgery	94	6.3	2	4.2	
Gynecology	85	5.7	3	6.3	
Vascular surgery	74	4.9	9	18.8	
Urologic surgery	66	4.4	7	14.6	
Traumatology surgery	51	3.4	1	2.1	
Pediatric surgery	47	3.1	0	0	
Head, neck, and breast surgery	30	2.0	1	2.1	

PAC = preanesthesia assessment clinic.

### 3.2. Reasons for case cancelations

**Figure 3.**
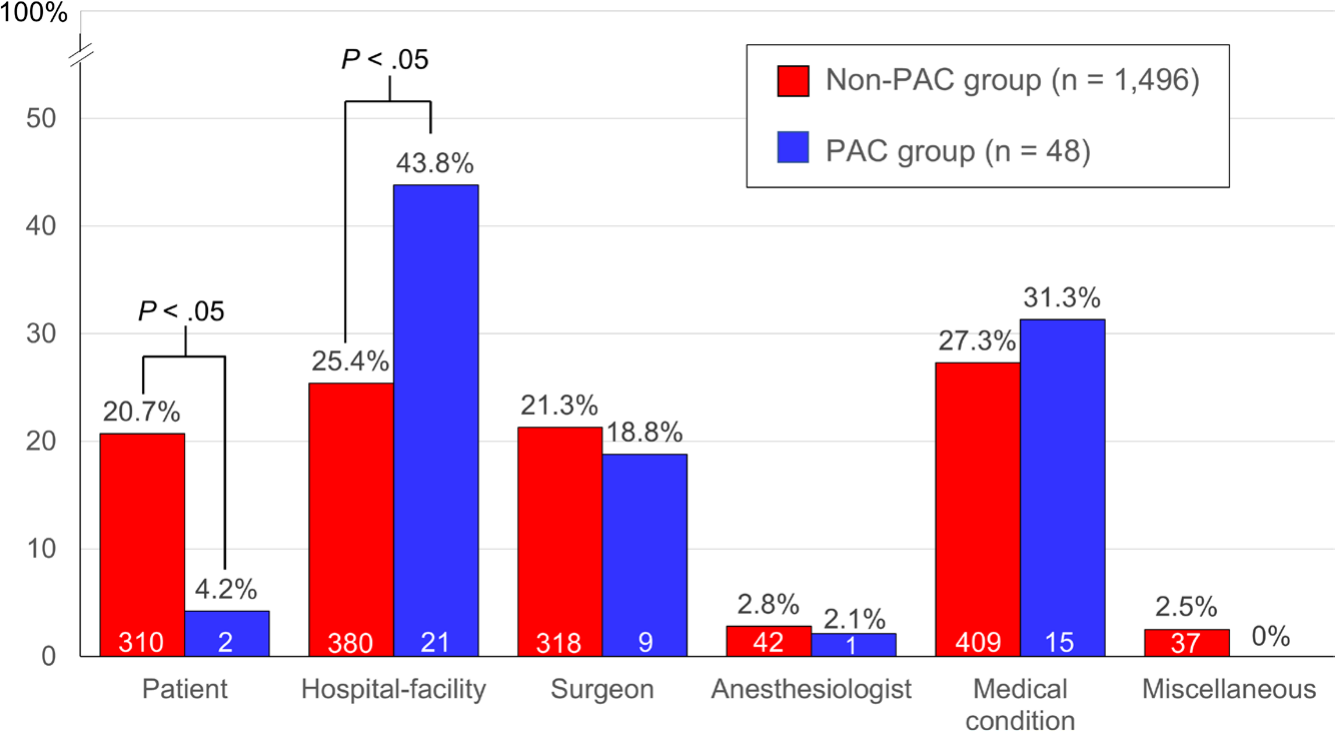
Reason categories for case cancelations. PAC = preanesthesia assessment clinic.

A comparison of the cancelation-reason categories of the PAC and non-PAC groups is depicted in Figure 3. Medical conditions were the most common cause of cancelation in the non-PAC group (27.3%), while hospital-facility issues were the most frequent reason in the PAC group (43.8%). The 3 most common reasons for both groups were hospital-facility issues, medical conditions, and surgeon issues. Anesthesiologist issues accounted for less than 3% of cases in both groups. There was a statistically significant difference in the case-cancelation frequencies by category of the PAC and non-PAC groups (*P* = .016). A post hoc analysis identified that the incidences of patient issues and hospital-facility issues of the PAC and non-PAC groups differed significantly (*P* < .05).

The reasons for cancelation in the PAC and non-PAC groups are detailed in Table 3. An improperly estimated case time was the leading cause of cancelation in both groups, accounting for approximately one-fifth of all elective case withdrawals. The second and third most frequent reasons for canceling in the non-PAC group were a changed line of surgical management (16.3%) and patient no-show (15.6%). In the PAC group, cardiovascular problems (14.6%) and ICU-bed unavailability (12.5%) ranked second and third.

**Table 3:** Reasons for case cancelations.

Cancellation reason	Non-PAC group (n = 1496)		PAC group (n = 48)	
	Number	%	Number	%
Patient issue				
Patient no-show	234	15.6	–	–
Patient refusal	72	4.8	2	4.2
Patient death	4	.3	–	–
Hospital-facility issue				
Improperly-estimated case time	255	17.0	11	22.9
ICU unavailability	56	3.7	6	12.5
Case bump	50	3.3	3	6.3
Equipment unavailability	8	.5	–	–
Hospital bed unavailability	9	.6	1	2.1
Blood products unavailability	2	.1	–	–
Surgeon issue				
Changed line of surgical management	244	16.3	6	12.5
Scheduling error	37	2.5	–	–
Surgeon unavailability	24	1.6	–	–
Inadequate preparation	13	.9	3	6.3
Anesthesiologist issue				
Preoperative drug error	28	1.9	1	2.1
Inadequate nil per os status	9	.6	–	–
Anesthesiologist refusal	5	.3	–	–
Medical condition				
Respiratory problem	124	8.3	4	8.3
Cardiovascular problem	84	5.6	7	14.6
Skin infection	43	2.9	–	–
Fever–unspecified	31	2.1	–	–
Gastrointestinal tract problem	26	1.7	–	–
Hematologic problem	25	1.7	–	–
Endocrine problem	20	1.3	1	2.1
Eye problem	15	1.0	1	2.1
Urogenital problem	15	1.0	–	–
Electrolyte imbalance	12	.8	–	–
Neurologic problem	10	.7	1	2.1
Gynecological problem	4	.3	1	2.1
Miscellaneous				
Health insurance problem	26	1.7	–	–
The reason could not be determined from records	7	.5	–	–
Data unavailability or missing data	4	.3	–	–

PAC = preanesthesia assessment clinic.

### 3.3. Cancellation-reason analysis in the PAC group

The cancelation reasons of the 48 patients in the PAC group were analyzed and classified as nonpreventable and potentially preventable. Seventeen cancelations (35.4%) were deemed nonpreventable (2 patient refusals, 3 case bumps, and 12 medical conditions). With the 12 nonpreventable medical conditions, 6 patients suffered clinical deteriorations of known medical conditions (3 cases of hypertension, 1 case of heart failure, 1 case of diabetes mellitus, and 1 case of asthma). Five other patients developed new acute illnesses shortly before surgery. The twelfth patient, a woman, developed a heart block after receiving a retrobulbar block; her operation was terminated.

The remaining 31 of the 48 cancelations (64.6%) were assessed as potentially preventable. They comprised cancelations due to improperly estimated case times (11), ICU-bed unavailability (6), hospital-bed unavailability (1), changed line of surgical management (6), inadequate preparations (3), preoperative drug errors (1), and medical conditions (3). With the 3 potentially preventable medical conditions, 2 patients had cardiac abnormalities not detected by the PAC (left ventricular aneurysm and abnormal cardiac murmur), and one had a menstrual cycle. Table 4 summarizes the details of the 15 cases that were canceled because of nonpreventable and potentially preventable medical conditions.

**Table 4:** Information on preanesthesia assessment clinic patients canceling because of a medical condition.

No.	Sex	Age	Procedure	OPD/IPD	Cancellation reason	Information	Preventable
1	Male	74	Transurethral resection of the prostate	IPD	Cardiovascular problem	Left ventricular aneurysm	Potentially
2	Male	76	Prostatectomy	IPD	Cardiovascular problem	Pansystolic murmur	Potentially
3	Female	40	Exploratory laparotomy	IPD	Gynecological problem	Having menstrual cycle	Potentially
4	Female	72	Microforaminotomy	IPD	Cardiovascular problem	Hypertensive urgency	No
5	Female	78	Arteriovenous fistula	IPD	Cardiovascular problem	Hypertensive urgency	No
6	Female	36	Arteriovenous bypass graft	IPD	Cardiovascular problem	Hypertensive urgency	No
7	Female	33	Mitral valve surgery	IPD	Cardiovascular problem	Congestive heart failure	No
8	Female	66	Pars plana vitrectomy	OPD	Cardiovascular problem	New right bundle branch block	No
9	Female	5	Eye exam	OPD	Respiratory problem	Upper respiratory tract infection	No
10	Female	5	Eye exam	OPD	Respiratory problem	Upper respiratory tract infection	No
11	Female	30	Exploratory laparotomy	IPD	Respiratory problem	Upper respiratory tract infection	No
12	Male	65	Ureteroscopic lithotripsy	IPD	Respiratory problem	Acute asthmatic attack	No
13	Female	83	Wound debridement	IPD	Neurologic problem	Lower limb weakness	No
14	Male	61	Posterior lumbar interbody fusion	IPD	Eye problem	Herpes zoster ophthalmicus	No
15	Female	58	Diastasis correction	IPD	Endocrine problem	Hyperglycemia	No

IPD = inpatient department, OPD = outpatient department.

One of the preoperative-drug-error cancelations was deemed a potentially preventable anesthesiologist issue. Although the patient was taking a high-dose corticosteroid as pemphigus therapy, he did not disclose its use when the PAC staff reviewed his medications. He only disclosed the corticosteroid use on the day of the surgery. The operation was postponed to evaluate his risk for adrenal insufficiency and the need for perioperative glucocorticoid administration.

## 4. Discussion

The incidence of cancelations for patients who attended the PAC before surgery (the PAC group) was 0.9%. This proportion was significantly lower than the 5.9% for those who underwent surgery without a PAC consultation (the non-PAC group). The overall cancelation incidence of scheduled elective procedures of 5.09% is consistent with findings at other university hospitals in Thailand (3.9–6.4%).^[17,18]^

Our findings are consistent with an Irish study that found that visiting a PAC significantly reduced surgical case cancelations.^[6]^ Research at a university hospital in the United States revealed that patients who visited its PAC had nearly 50% fewer cancelations. Another prospective study at a large Chinese medical center found a dramatic reduction in case cancelations (from 7.6% in the non-PAC group to 0% in the PAC group).^[10,19]^ Such findings confirm PAC consultations’ significant benefit of reducing case cancelations on the day of surgery.

Our study found that the case-cancelation frequencies of the non-PAC and PAC groups differed significantly by category, particularly for patient issues and hospital-facility issues. The study conducted in Ireland concluded that the establishment of a PAC had no statistically significant impact on cancelations related to patient issues.^[6]^ However, our study showed a contrasting result, with patients in the PAC group having approximately a fifth of the proportional level of case cancelations attributable to patient issues as the non-PAC group. This substantial difference can be explained by the significant reduction in the patient no-show rate of our study’s PAC group to 0%.

Concerning the hospital-facility-issue category, the case-cancelation frequency was markedly higher for the PAC group, 43.8%, than for the non-PAC group (25.2%). The indicated patients referred to the PAC were probably those who needed major surgery or had an ASA III or higher; thus, a lack of hospital facilities, such as an ICU bed, was more impactful. A contrasting result – a significant reduction in hospital-related cancelations in the PAC group – was reported by the Irish study. However, its authors explained that this occurrence was not attributable to their PAC but was likely due to a marked increase in their hospital bed availability during the study period.^[6]^

The most common reason for cancelation in the non-PAC and PAC groups was improperly estimated case times, at approximately 20%. A similar result was demonstrated in systematic reviews that found that operating room unavailability was the principal reason for day surgery cancelations.^[20,21]^ Our findings indicated that the PAC service could not improve this type of cancelation. Moreover, other studies reported that another major cause was patient issues (for instance, no-shows and refusals),^[11,18]^ with such issues playing an important role in an ambulatory setting.^[4]^ Impressively, our study’s proportion of patient no-shows was dramatically lower in the PAC group (0%) than in the non-PAC group (15.6%). This finding affirms the effectiveness of our PAC in improving patient attendance, even in an outpatient setting.

The primary goal of our PAC was to optimize medical problems and reduce cancelations due to medical conditions. Nevertheless, our investigation determined that the proportions of medical condition-related cancelations in the non-PAC and PAC groups were comparable. An in-depth analysis of cancelations in the PAC group revealed that 11 of the 15 (73.3%) medical conditions were related to acute patient illnesses. Five of the patients developed new acute illnesses, whereas the other 6 patients experienced acute exacerbations, urgent/emergency situations, or sudden deteriorations stemming from underlying diseases. Even though the patients were optimized at the PAC, these acute patient illnesses developed shortly before surgery and were barely preventable. As a result, acute patient illness remained the common reason for cancelation. This finding is consistent with other studies, which reported that acute patient illness accounted for between 20% and 30% of all cancelations.^[8,20,21]^ To decrease the incidence of cancelations due to acute patient illness, patients may need to be scheduled for an additional PAC visit just a few days ahead of their scheduled surgery.

PAC visits are supposed to include a detailed review of all existing illnesses and uncover hidden conditions.^[14]^ Research elsewhere found that incomplete medical diagnoses and optimizations contributed most to avoidable cancelations^[22]^ Our investigation revealed room for improvement as there were 3 potentially preventable cases. Two of our patients had abnormal cardiac conditions that could have been addressed and managed when visiting the PAC but were not. Moreover, it was found that there was a female patient whose cancelation was likely preventable by her gynecologist. Either prescribing medication to control her menstrual period or planning a more appropriate surgery date was a possible solution. While a PAC has a role in preventing cancelations due to medical conditions, it seems that surgeons can more readily prevent some cancelations.

Furthermore, in the PAC group, anesthesiologist issues accounted for approximately 2% of canceled cases, consistent with earlier findings.^[18,20]^ Inadequate nil-per-os and inappropriate management of antiplatelet and anticoagulant medications are common cancelation reasons worldwide. However, no cases in our PAC group were canceled for these reasons.^[21,23]^ This finding demonstrated the PAC’s capability for providing preoperative patient education for both inpatients and outpatients.

This study has strengths. It is the first to evaluate the impact of an anesthesiologist-led PAC on the incidence of elective case cancelations at a Thai quaternary hospital. Surgery cancelations in the PAC group due to medical conditions and anesthesiologist issues were also explored to determine their preventability. However, there are some study limitations. First, it was retrospective, so the information regarding reasons for cancelation was retrieved entirely from medical records. Second, although there were structured indications for PAC visits, decisions to refer surgical patients to the clinic were made by surgeons; consequently, some biases might exist. Third, some advantages of a PAC were not investigated, for example, minimizing unnecessary testing, decreasing subspecialty consultations, and reducing preoperative hospital stays. Last, as the research was conducted at a single university center where the preoperative clinic was well established, the outcomes might not be generalizable to other healthcare settings.

The PAC mainly aims to optimize medical conditions to enhance patient outcomes and avoid medical condition-related cancelations. It is a powerful approach to reducing elective cancelations on the day of surgery. However, more efficient and effective hospital-facility resource management and surgery scheduling systems could also be potential solutions, and research should be conducted to fully establish their advantages. Moreover, a successful perioperative care pathway requires effective, well-structured, multidisciplinary collaboration and communication. Finally, regular staff and system performance evaluations are necessary to promote quality improvement.

## 5. Conclusions

Our PAC could effectively reduce the incidence of elective procedure cancelations on the day of surgery. The cancelation rate for patient issues was significantly lower in the PAC group. The top cancelation-reason category was hospital-facility issues for patients who visited the PAC, whereas it was medical conditions for those who did not. Most medical conditions in the PAC group were nonpreventable acute patient illnesses. Nevertheless, some potentially preventable cases could have benefited from more rigorous evaluation by the PAC.

## Acknowledgments

The authors thank Chusana Rungjindamai, a research assistant, Department of Anesthesiology, for summarizing the documents; and Pornprapa Thiansri, an administrative officer, Department of Anesthesiology, for collecting data.

## Author contribution

**Conceptualization:** Mingkwan Wongyingsinn.

**Data curation:** Mingkwan Wongyingsinn.

**Formal analysis:** Mingkwan Wongyingsinn.

**Investigation:** Pitchapa Wongcharoen, Mingkwan Wongyingsinn.

**Methodology:** Mingkwan Wongyingsinn.

**Project administration:** Mingkwan Wongyingsinn.

**Supervision:** Mingkwan Wongyingsinn.

**Validation:** Wariya Vongchaiudomchoke, Pitchapa Wongcharoen, Mingkwan Wongyingsinn.

**Visualization:** Wariya Vongchaiudomchoke, Pitchapa Wongcharoen, Mingkwan Wongyingsinn.

**Writing – original draft:** Wariya Vongchaiudomchoke.

**Writing – review & editing:** Wariya Vongchaiudomchoke, Mingkwan Wongyingsinn.
